# Advanced Chemical Computing Using Discrete Turing Patterns in Arrays of Coupled Cells

**DOI:** 10.3389/fchem.2020.559650

**Published:** 2020-10-29

**Authors:** František Muzika, Lenka Schreiberová, Igor Schreiber

**Affiliations:** Department of Chemical Engineering, University of Chemistry and Technology, Prague, Czechia

**Keywords:** chemical computing, discrete Turing patterns, coupled cells, bifurcation analysis, glycolytic oscillations

## Abstract

We examine dynamical switching among discrete Turing patterns that enable chemical computing performed by mass-coupled reaction cells arranged as arrays with various topological configurations: three coupled cells in a cyclic array, four coupled cells in a linear array, four coupled cells in a cyclic array, and four coupled cells in a branched array. Each cell is operating as a continuous stirred tank reactor, within which the glycolytic reaction takes place, represented by a skeleton inhibitor-activator model where ADP plays the role of activator and ATP is the inhibitor. The mass coupling between cells is assumed to be operating in three possible transport regimes: (i) equal transport coefficients of the inhibitor and activator (ii) slightly faster transport of the activator, and (iii) strongly faster transport of the inhibitor. Each cellular array is characterized by two pairs of tunable parameters, the rate coefficients of the autocatalytic and inhibitory steps, and the transport coefficients of the coupling. Using stability and bifurcation analysis we identified conditions for occurrence of discrete Turing patterns associated with non-uniform stationary states. We found stable symmetric and/or asymmetric discrete Turing patterns coexisting with stable uniform periodic oscillations. To switch from one of the coexisting stable regimes to another we use carefully targeted perturbations, which allows us to build systems of logic gates specific to each topological type of the array, which in turn enables to perform advanced modes of chemical computing. By combining chemical computing techniques in the arrays with glycolytic excitable channels, we propose a cellular assemblage design for advanced chemical computing.

## Introduction

Living cells can be considered as autonomous systems, which developed through evolution into energetically efficient forms capable of analysis of the environment to find sources of energy and material for maintenance, metabolism, and replication. Their subsystem for environmental analysis requires an intracellular signaling network, such as sensor/receptor-repressor system of *Saccharomyces cerevisiae* for glucose detection based on kinases (Snowdon and Johnston, 2016), or signaling network based on MAPkinases (Sauro and Kholodenko, 2004; Hadač et al., 2017). In multicellular organisms, due to cell differentiation, a signaling network system developed into specialized signaling cells, the neurons, which form a network of cells creating the signaling system of multicellular organisms. To show the energy efficiency of such cells we highlight a human neuron. It consumes in average 8 × $10^{-17}\frac{mol}{cell\cdot s}$ to 4 × $10^{-16}\frac{mol}{cell\cdot s}$ of glucose and 1 × $10^{-17}\frac{mol}{cell\cdot s}$ to 1 × $10^{-15}\frac{mol}{cell\cdot s}\;$of oxygen (McMurtrey, 2016). Neurons display high arithmetic and pattern recognition performance, when integrated into network (Majaj et al., 2015). Around 40 percent of consumed energy is used for basal energetic consumption and the rest is used to form and send currents among individual neurons, therefore 60 percent of consumed energy is used for data processing (Engl and Attwell, 2015).

Since the network of signaling neurons is energetically efficient, it is a source of ideas and techniques for building artificial signaling systems called neural networks. Examples of physically constructed neural networks include programmable resistive elements (memristors) (Howard et al., 2014); complementary metal–oxide–semiconductors (CMOS), which Shen et al. used to construct a Darwin neural processing unit with 2,048 neurons and power consumption ~60 mW (Shen et al., 2016); magnetic spin switches in 3D/2D architecture (Roy et al., 2014); a pattern recognition technique, based on network of bistable rectors (Hjelmfelt and Ross, 1993; Hjelmfelt et al., 1993); network of gel droplets containing BZ reaction system (Holley et al., 2011; Górecki et al., 2014) and neuron-like units constructed using excitable channels (Górecka and Górecki, 2006).

Neural network processors can be constructed also from biological materials, for example: substitution of neuron units with DNA cascades (Qian et al., 2011); techniques combining DNA assays and transistors based on natural fluorescence (Lue and Fang, 2008); substitution of neuron units by three types of oscillators under batch conditions (Kim and Winfree, 2011). The neural network can be substituted by a lattice of excitable cells capable of signal addition, subtraction and conduction (Adamatzky, 1998). It can be constructed as a model of linear 3D neural network, using N layers of lattices, where (X-1)-th layer is connected to X-th layer and the X-th layer is connected to (X+1)-th layer, where X = 1, …,N. This theoretical work was followed by an experimental 2D lattice of liquid crystals to perform logic calculations (Adamatzky et al., 2011).

In this work, we explore a simple substitute for a neural network that performs chemical computing by utilizing discrete Turing patterns occurring in a network of mass coupled chemical cells with an autocatalytic reaction. From a theoretical point of view, the origin of biological morphogenesis was proposed by A. Turing (Turing, 1952) as manifestation of spatiotemporal patterns arising due to reaction-diffusion in cyclic arrays of coupled cells or in continuous tissue. Although his theory was shown not to be valid in general (Wolpert, 1969; Kerszberg and Wolpert, 1998), it does seem to apply in special cases, such as functional development of a brain tissue (Garzón-Alvarado et al., 2011), development of digits of mice (Bagudu et al., 2012) or during Saccharomyces cerevisiae cell polarization (Kozubowski et al., 2008; Giese et al., 2017). The key condition for occurrence of Turing patterns is a synergy of input, output, autocatalysis, inhibition, and diffusion. For spontaneous occurrence, diffusion parameters of specific components have to be set to exhibit short range activation and long range inhibition (Meinhardt and Gierer, 1974, 2000; Kondo and Miura, 2010), in other words, diffusion coefficient of an inhibitor has to be higher than diffusion coefficient of an activator. In an array of cells, the Turing instability gives rise to a non-uniform discrete stationary concentration profile throughout the array. A non-uniform concentration profile can thus be viewed as a discrete Turing pattern. In contrast, within a continuous tissue, the Turing instability leads to smooth spatiotemporal structures such as labyrinth (Rudovics et al., 1999; Asakura et al., 2011), dots (Ouyang et al., 1995; Rudovics et al., 1999; Vanag and Epstein, 2001), stripes (Ouyang et al., 1995), hexagons (Horvath et al., 2009; Asakura et al., 2011), or helical patterns in cylindrical layers (Bánsági and Taylor, 2015).

In a system of coupled oscillators, discrete Turing patterns often coexist with oscillations, In particular, they were shown to coexist by Bar-Eli (1984) and Vastano et al. (1987) for equal transport rate coefficient of activator and inhibitor. Such a system can be carefully perturbed to induce transition from oscillations to a discrete Turing pattern. Early experimental research was performed using the BZ oscillatory system, where a membrane between cells was substituted by valves (Crowley and Epstein, 1989) or peristaltic pumps (Bar-Eli and Reuveni, 1985; Dolník and Marek, 1988; Yoshimoto et al., 1993). Following these findings, in our previous work we examined the case of equal transport rate coefficients of activator and inhibitor using a core model of (Goldbeter and Moran, 1984) glycolysis as an oscillatory reaction. We identified coexisting discrete Turing patterns in linear arrays of two and three coupled cells (Muzika and Schreiber, 2013; Muzika et al., 2014) and applied targeted perturbations to perform basic logical functions. In our experimental research, we substituted membrane by a reciprocal peristaltic pumping to form a cyclic array of four coupled subsystems where the reaction of yeast extract and D-glucose took place (Muzika et al., 2016). In agreement with our theoretical predictions we found coexistence of discrete Turing patterns with uniform oscillations and were able to apply specific perturbations, inducing transition between discrete Turing patterns and uniform oscillations.

The paper is organized as follows. In section Model we provide details of the glycolytic model and formulate equations describing arrays of coupled cells with an arbitrary topology. In section Stability And Bifurcation Analysis the analysis of stability and bifurcations of stationary states is used to construct bifurcation diagrams for arrays of three and four coupled cells at various fixed ratios of transport rate coefficients of ATP and ADP and identify various types of discrete Turing patterns, their occurrence, coexistence, and stability within a 2D parameter space. By choosing specific regions with occurrence of multiple discrete Turing patterns in arrays of three and four coupled cells with various topology (linear, cyclic, T-shaped) and for various ratios of transport rate coefficients, one-parameter diagrams are chosen to provide a more detailed insight. Finally, section Chemical Computing Devices is focused on studying various aspects of chemical computing. By creating a system of precisely targeted and precisely timed perturbations to induce transitions between discrete Turing patterns and oscillations, we discuss a tautology/contradiction gate, advanced logic functions gates, and advanced cellular assemblage design.

## Model

Glycolysis is one of the oldest and most common biochemical oscillatory reaction. Its purpose is to release energy from carbohydrates, which a cell synthesizes during photosynthesis or which a cell consumes from an external supply. A mathematical model of the yeast glycolytic reaction chain proposed by Hynne et al. (2001) consists of 24 reactions. It incorporates an autocatalytic enzymatic reaction mediated by phosphofructokinase and it also contains negative feedback enzymatic reactions from the lower part of the glycolytic chain catalyzed by pyruvate kinase and phosphoglycerate kinase. To analyze dynamic behavior in the arrays of mass-coupled cells, it is convenient to reduce the model involving entire glycolytic reaction chain into a core model retaining only the three aforementioned positive and negative feedback reactions. Therefore, for our analysis of bifurcations and stability of stationary states, the core model proposed by Goldbeter and Moran (1984) is used.

From an experimentalist viewpoint both feedback reactions can be regulated through temperature adjustments and also through the level of pH, where synergic effect with fructose 2,6 bisphosphate occurs between pH = 9 and pH = 5. In this range, the activity of phosphofructokinase is increased due to a decreased energy consumption to create bonds (Deville-Bonne et al., 1991; Tlapak-Simmons and Reinhart, 1998). Phosphofructokinase can be stimulated by addition of glycolytic metabolites or by addition of other components. In particular, hydrocarbonate can increase the activity of phosphofructokinase three times. *In vivo* experiments have shown that the addition of hydrocarbonate increased motility of sperm cells (Hereng et al., 2014) due to increased ATP-pool levels. We observed and used the same effect to increase activity of phosphofructokinase in our experimental research of discrete Turing patterns (Muzika et al., 2016). Phosphofructokinase can be inhibited by addition of: (1) ATP by up to 95%, (2) citrate by up to 60%, (3) PEP by up to 50%, (4) fructose 6-phosphate by up to 70%, and (5) phosphoglycerate by up to 60% (Mediavilla et al., 2007). These positive and negative effects provide rationale for modifying corresponding rate coefficients in the core model of glycolysis to a considerable extent. Coupling of cells in multicellular organisms is realized via gap junctions or in the case of artificial cellular assemblages via artificial membranes or artificial ports/junctions. Correspondingly, we add linear diffusion terms to the core model, creating the following model of N coupled cells with various topologies:

(1) $$\begin{array}{l} \frac{dx_{i}}{dt}=f_{x}\left(x_{i},y_{i}\right)+qk_{ADP}\overset{N}{\underset{j=1}{\sum }}\delta_{ij}\left(x_{j}-x_{i}\right)+p_{i}\left(t\right),\\ \frac{dy_{i}}{dt}=f_{y}\left(x_{i},y_{i}\right)+k_{ADP}\sum_{j=1}^{N}\delta_{ij}\left(y_{j}-y_{i}\right), \end{array}$$

(2) $$\begin{array}{l} f_{x}=\nu +\sigma_{inh}\frac{y_{i}^{n}}{M^{n}+y_{i}^{n}}-\sigma_{M}\frac{x_{i}\left(1+x_{i}\right){\left(1+y_{i}\right)}^{2}}{L+{\left(1+x_{i}\right)}^{2}{\left(1+y_{i}\right)}^{2}}\;,\\ f_{y}=\phi \sigma_{M}\frac{x_{i}\left(1+x_{i}\right){\left(1+y_{i}\right)}^{2}}{L+{\left(1+x_{i}\right)}^{2}{\left(1+y_{i}\right)}^{2}}-k_{S}y_{i}-\phi \sigma_{inh}\frac{y_{i}^{n}}{M^{n}+y_{i}^{n}}\;,\\ \;i=1,\ldots ,N, \end{array}$$

where {δ_ij_} is a non-negative structural matrix specifying the topology of the array. In the special case of 1D (non-cyclic) chain:

δ_*ij*_ = δ_*ji*_ = 1 *for j* = *i* − 1; *i* = 2, …, *N*,

δ_*ij*_ = 0 otherwise,

and for a cyclic chain:

δ_*ij*_ = δ_*ji*_ = 1 *for j* = *i* − 1; *i* = 2, …, *N*,

δ_1*N*_ = δ_*N*1_ = 1,

δ_*ij*_ = 0 otherwise.

By properly choosing {δ_ij_}, more complex topologies, such as T-shaped array can be defined.

The symbols *x*_*i*_ and *y*_*i*_ represent dimensionless concentrations of ATP and ADP in the i-th cell, respectively. The function *p*_*i*_*(t)* represents perturbation of i-th cell by ATP, see section Chemical Computing Devices for more detail. The parameters are as follows: *M* is Michaelis constant; ν represents ATP uptake rate; *n* represents Hill coefficient; ϕ is the ratio of dissociation constants of ATP and ADP; *L* represents allosteric constant specifying affinity of the PFK conformation to the reactive state rather than non-reactive conformation (Monod et al., 1965); *k*_*s*_ represents removal rate coefficient of ADP; σ_*M*_ represents rate coefficient of autocatalysis; σ_*inh*_ represents rate coefficient of inhibition; *q* represents ratio of the transport coefficient of ATP relative to ADP; *k*_*ADP*_ represents transport coefficient of ADP between each pair of coupled cells. The following parameters are set according to Goldbeter and Moran (1984): ϕ = *1*, ν = 1.84 s^−1^, *L* = 5 × 10^6^, *n* = 4, *M* = 10, *k*_*s*_= 0.06 s^−1^. There is a unique stationary state in one cell that, depending on the two remaining kinetic parameters σ_*M*_ and σ_*inh*_, is either stable or undergoes an oscillatory instability via a Hopf bifurcation. Below, we treat σ_*M*_ and σ_*inh*_ as adjustable, as well as the coupling parameters *k*_*ADP*_ and *q*. We use these four free parameters to construct various bifurcation diagrams and thus demonstrate their effect on occurrence of discrete Turing patterns and their overlap with homogeneous periodic oscillations.

## Stability and Bifurcation Analysis

### Bifurcation Scenarios

For the analysis of stationary and dynamic behavior of the glycolytic oscillatory reaction in arrays with various topology we use the program CONT (Kubíček and Marek, 1983; Kohout et al., 2002). We chose such a parameter setup that the system exhibits either a unique stable stationary state or unique stable limit cycle in one cell avoiding thus the region of birhythmicity (Goldbeter and Moran, 1984). These two basic regimes translate in the context of arrays into a uniform stationary state and uniform oscillations. However, their stability generally depends on the coupling strength. Under parameter settings used in this work, the stable uniform stationary state may lose stability either via a Hopf bifurcation leading to stable uniform oscillations or via a symmetry breaking bifurcation leading to stable discrete Turing patterns (i.e., stable non-uniform stationary states). In addition, symmetry breaking bifurcation may occur also from unstable uniform stationary states. On the other hand, symmetry breaking bifurcation from the uniform oscillations was never observed. These so-called *primary* bifurcations from the uniform stationary states are complemented by *secondary* bifurcations from the non-uniform stationary states, which include limit point bifurcations (folds), secondary Hopf bifurcations as well as secondary symmetry breaking bifurcations. Any Hopf bifurcation curve in a two-parameter plane is either a closed curve or it terminates when touching a curve of limit point bifurcation at the Bogdanov-Takens codimension one point. Also, two Hopf bifurcation curves may intersect at the point of double Hopf bifurcation; additionally, there are other types of singularity points, which we do not mention here. Details of all these transitions depend on the particular array topology and may be quite involved as described below.

**Figure 1:**
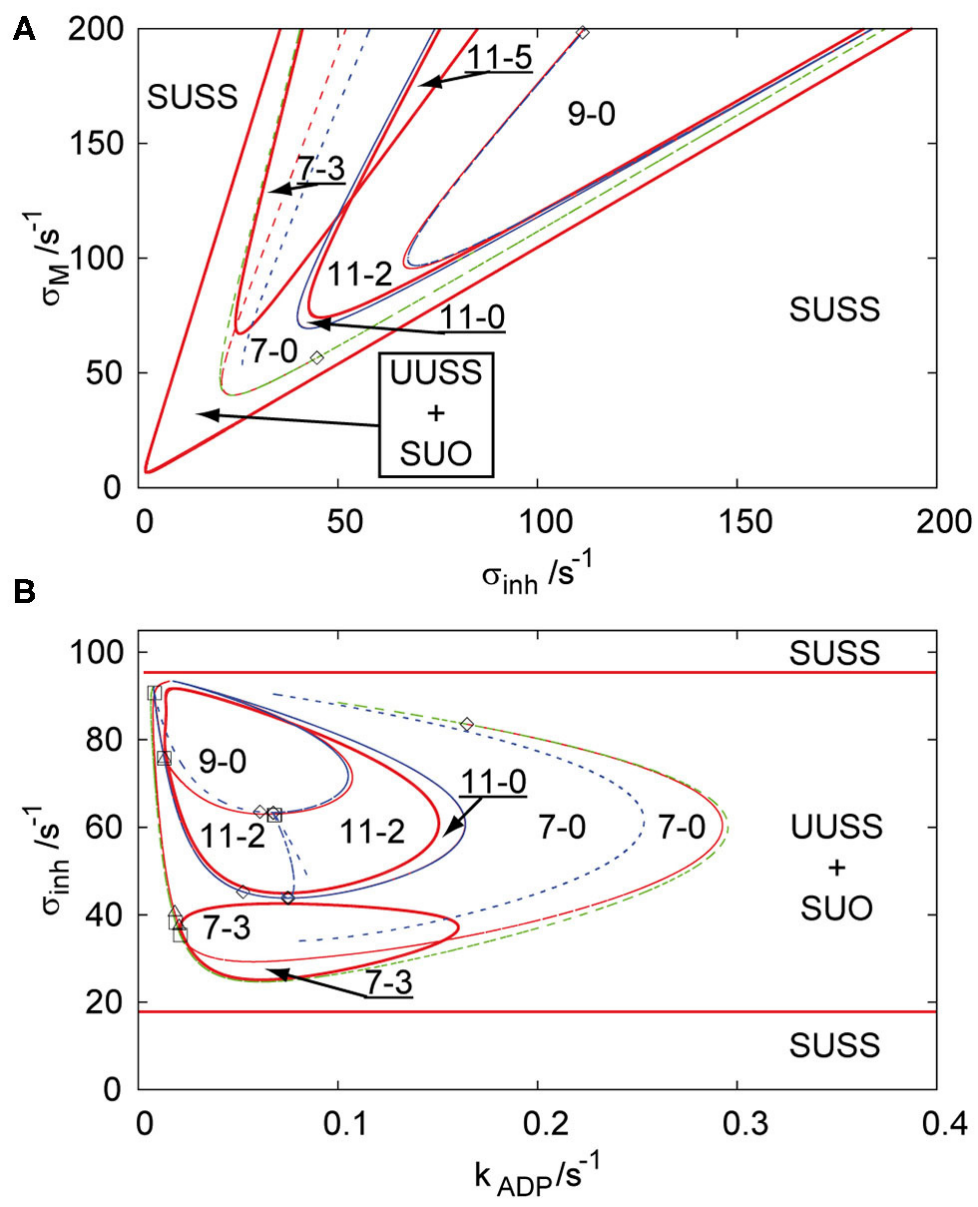
Bifurcation diagram for three coupled cells in cyclic array, *q* = 1, in planes: **(A)** σ_*inh*_ and σ_*M*_, *k*_*ADP*_ = 0.1 s^−1^; **(B)** σ_*inh*_ and *k*_*ADP*_, σ_*M*_ = 100 s^−1^. Red curve—Hopf bifurcation curve; blue curve—symmetry breaking bifurcation curve; green curve—limit point curve; full line—change of stability across the line, dashed curve—no change of stability across the line; empty square—intersection point of Hopf bifurcation/symmetry breaking bifurcation/limit point curves; empty diamond—Bogdanov-Takens bifurcation point; empty triangle—double Hopf bifurcation point; SUSS, stable uniform stationary state; UUSS, unstable uniform stationary state; SUO, stable uniform oscillations. Each parameter region is marked by a pair of numbers m,n, where m is total number of stationary states and n is the number of stable stationary states.

In our previous work, an array of two coupled cells (Muzika et al., 2014) and a linear array of three coupled cells (Muzika and Schreiber, 2013) were described through bifurcation diagrams in the parameter plane of σ_*M*_ and σ_*inh*_ and in the parameter plane of σ_*inh*_ and *k*_*ADP*_ at fixed *q*. In the following we use the same parameter planes. The region of stable uniform stationary state (SUSS) in Figures 1A,B is a region that all arrays with *q* ≤ 1 share throughout all the topologies, because it does not depend on *k*_*ADP*_. The region of unstable uniform stationary state (UUSS) and simultaneously stable uniform oscillations (SUO) contains subregions of coexisting stable or unstable non-uniform stationary states (discrete Turing patterns) for all arrays with *q* ≤ 1. The regions of Turing patterns are described with numbers in the format a-b, where the first number defines the number of stationary states and the second number defines the number of stable stationary states. Figure 1A shows that with increasing positive feedback rate coefficient σ_*M*_, two triplets of unstable symmetric non-uniform patterns occur through a primary symmetry breaking bifurcation from an unstable uniform stationary state and the limit point curve delimits the region of their occurrence (region 7-0). Three of these unstable non-uniform patterns become stabilized (region 7-3) at ≈ σ_*M*_ = 70 s^−1^ by a Hopf bifurcation (full red curve), which is called *secondary* stabilization. At higher inhibition rate coefficient σ_*inh*_, four new non-uniform stationary states occur from two non-uniform branches via secondary symmetry breaking bifurcation (from non-uniform symmetric to non-uniform asymmetric) creating the region 11-0. With increasing autocatalytic rate coefficient, two of the unstable non-uniform stationary states are stabilized by a secondary Hopf bifurcation curve (region 11-2). Further simultaneous increase of both σ_*M*_ and σ_*inh*_ leads to another Hopf bifurcation curve, which destabilizes the stable non-uniform patterns (region 9-0) again, creating a U-shaped region of stable Turing patterns (region 11-2). These two regions of stable non-uniform stationary states (region 11-2 and region 7-3) intersect each other above ≈ σ_*M*_ = 125 s^−1^ creating a parameter region with five stable non-uniform stationary states (region 11-5). Figure 1B represents the parameter plane of σ_*inh*_ and *k*_*ADP*_ at σ_*M*_ = 100 s^−1^. This bifurcation diagram does not possess the intersection of both regions of stable non-uniform stationary states (region 7-3 and region 11-2 merging to region 11-5 in Figure 1A), instead it shows a disc (region 7-3) and two other embedded disks (region 11-2 and region 9-0) delimited by Hopf bifurcations with the region in-between them representing stable Turing patterns (region 11-2).

**Figure 2:**
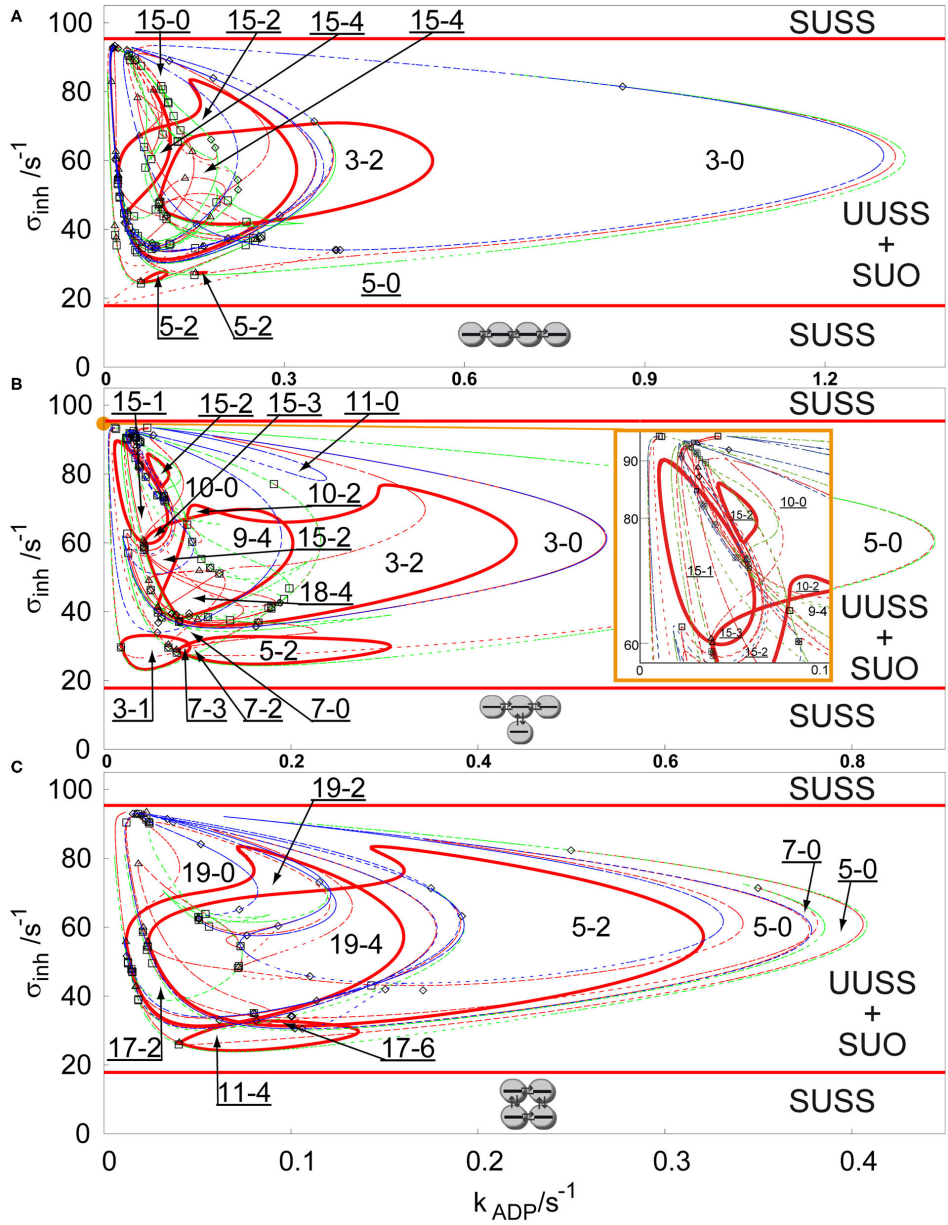
Comparison of bifurcation diagram four coupled cells in a parameter plane σ_*inh*_ and *k*_*ADP*_, *q* = 1, σ_*M*_ = 100 s^−1^ in: **(A)** linear array, **(B)** T-shaped array, **(C)** cyclic array. Red curve—Hopf bifurcation curve; blue curve—symmetry breaking bifurcation curve; green curve—limit point curve; full line—change of stability across the line, dashed curve—no change of stability across the line; empty square—intersection point of Hopf bifurcation/symmetry breaking bifurcation/limit point curves; empty diamond—Bogdanov-Takens bifurcation point; empty triangle—double Hopf bifurcation point; SUSS, stable uniform stationary state; UUSS, unstable uniform stationary state; SUO, stable uniform oscillations. Each parameter region is marked by a pair of numbers m,n, where m is total number of stationary states and n is the number of stable stationary states. For clarity, the upper-left corner region with many overlapping curves is magnified and displayed in the inset on the right.

For more complex topologies of arrays, bifurcation diagrams abound with curves of Hopf bifurcation, symmetry breaking bifurcation, and limit points, therefore we choose to show only the bifurcation diagrams for three additional types of arrays in the parameter plane σ_*M*_ and *k*_*ADP*_. Figure 2 shows three bifurcation diagrams for an array of four coupled cells with *q* = 1 with the following topologies: linear, T-shaped and cyclic. As in the previous case, at a first glance, the salient features of the diagrams are the disc-like regions of stable non-uniform stationary states (discrete Turing patterns) delimited by red curves of secondary Hopf bifurcation, which are coexisting with the stable uniform oscillations in the region delimited by two parallel lines of primary Hopf bifurcation. The region of non-uniform stationary states in Figure 2A (linear array) is the widest region of Figure 2 and contains up to 15 coexisting stationary states. Two non-uniform stationary states occur from a primary symmetry breaking bifurcation creating region 3-0, which is partly delimited by a limit point curve, where the number of non-uniform stationary states is doubled creating the region 5-0. Secondary Hopf bifurcation curves (full red curves) stabilize the unstable non-uniform stationary states delimiting the region 3-2 and the very small region 5-2. With decreasing *k*_*ADP*_ four new pairs of non-uniform stationary states occur and two of them are doubled by a limit point curve, therefore creating 15 stationary states. Two of them are secondarily stabilized creating the region 15-2 and where the region 3-2 intersects with 15-2, they create the region 15-4. Another region delimited by secondary symmetry breaking curve and limit point curve overlaps with this dense region and a small inner part of it is again secondarily stabilized by a Hopf bifurcation curve to form region 5-2.

The bifurcation diagram for a T-shaped array is shown in Figure 2B. The region 5-0 is much wider than in the case of the linear array, while the region 3-0 is narrower. The Hopf bifurcation curve (full red curve) secondarily stabilizes a subregion of 3-0 into the region 3-2. Another secondary Hopf bifurcation curve delimits the closed region 5-2 embedded in the region 5-0. Both cases can be observed in the case of the linear array, but the region 5-2 is much wider now. It is also intersected by another limit point curve, adding two unstable non-uniform stationary states creating the small region 7-2 and one stable and one unstable non-uniform stationary state creating the small region 7-3. The primary region 3-0 is further intersected with branch point and limit point curves, where the most interesting are two regions 15-2, which also occur in the case of the linear array, and the region, where stabilizing Hopf bifurcation curves intersect, creating the regions 18-4 and 9-4. The bifurcation diagram for the cyclic 4-array is shown in Figure 2C. The region 3-0 is missing, however due to intersections of regions narrowed by a limit point curves, there is a wide region 5-0 with small overlap 7-0. Unstable non-uniform stationary states are again stabilized by a secondary Hopf bifurcation curve creating a region 5-2. Then the whole region 5-0 is intersected by several symmetry breaking bifurcation curves creating up to 19 stationary states (region 19-0), which are stabilized by a Hopf bifurcation curve to form the region 19-2 and then the region intersects with 5-2 to form the region 19-4. This region intersects also with another region 11-4, creating the region 17-6 with the largest number of stable Turing patterns.

**Figure 3:**
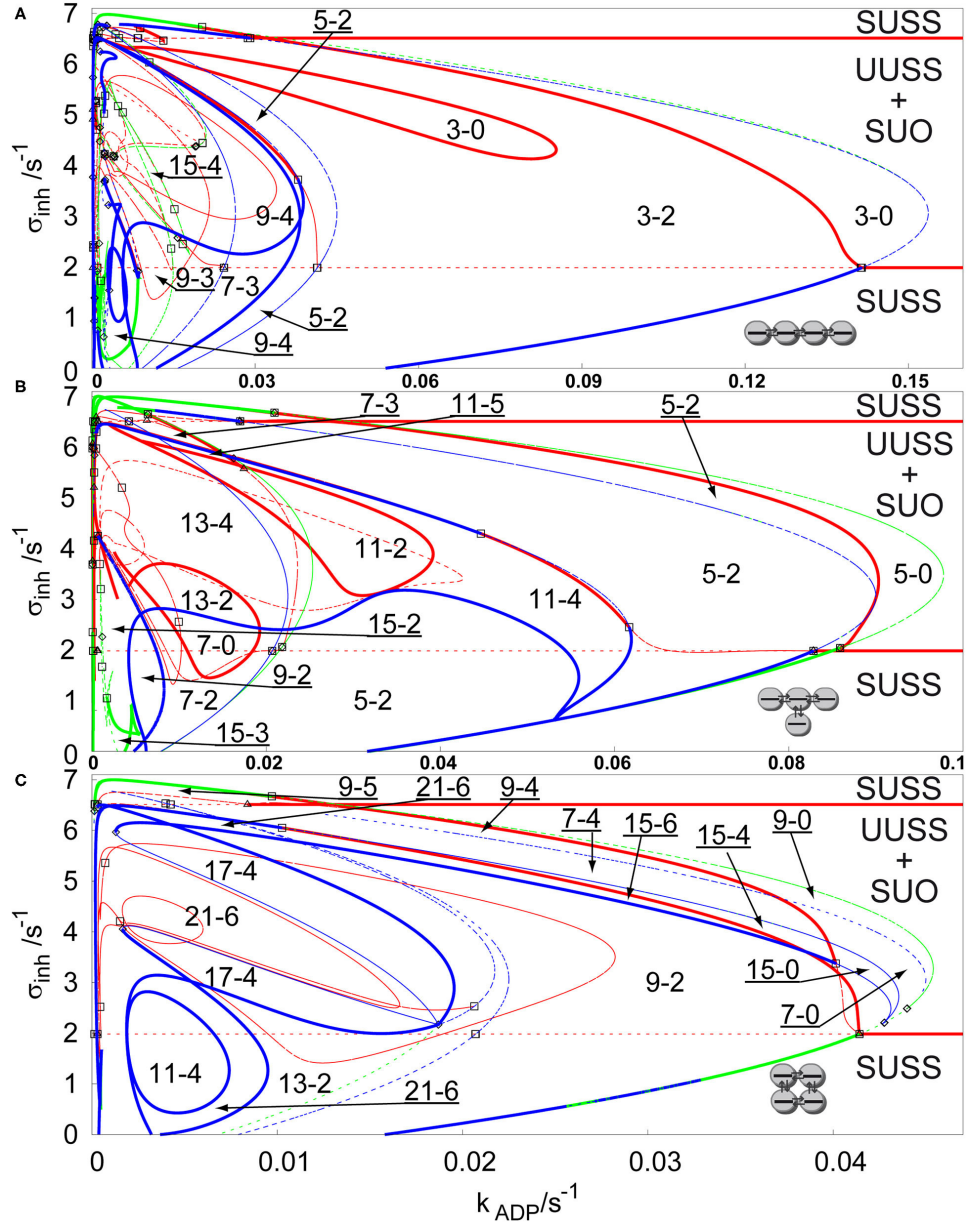
Comparison of bifurcation diagrams of four coupled cells in a parameter plane σ_*inh*_ and *k*_*ADP*_, *q* = 100, σ_*M*_ = 10 s^−1^ in: **(A)** linear array, **(B)** T-shaped array, **(C)** cyclic array. Red curve—Hopf bifurcation curve; blue curve—symmetry breaking bifurcation curve; green curve—limit point curve; full line—change of stability across the line, dashed curve—no change of stability across the line; empty square—intersection point of Hopf bifurcation/symmetry breaking bifurcation/limit point curves; empty diamond—Bogdanov-Takens bifurcation point; empty triangle—double Hopf bifurcation point. Each parameter region is marked by a pair of numbers m,n, where m is total number of stationary states and n is the number of stable stationary states.

For *q* > 1, a stable non-uniform stationary state may emerge directly via a primary symmetry breaking bifurcation from the stable uniform stationary state, which leads to spontaneous occurrence of Turing patterns as well-known from many experiments in spatially extended reactors (Castets et al., 1990; Ouyang et al., 1995; Rudovics et al., 1999; Dolník et al., 2001; Sanz-Anchelergues et al., 2001; Asakura et al., 2011). To ensure sufficient conditions for spontaneous occurrence of Turing patterns, we set *q* = 100 and σ_*M*_ = 10 s^−1^, while the other parameters remain, as they were proposed by Goldbeter and Moran (1984). We are comparing the previously discussed three types of arrays in Figure 3. It is clearly seen that the regions of Turing patterns now extend below the lower primary Hopf bifurcation line for each topology occurring via supercritical or transcritical branch point curves for sufficiently low inhibiton rate coefficients σ_*inh*_ and disappearing via subcritical or superscritical symmetry breaking bifurcation curves at σ_*inh*_ ≈ 7 s^−1^.Notice that σ_*M*_ = 10 s^−1^ (ten times lower than for case of *q* = 1), which causes the non-uniform patterns to occur in the region within the *k*_*ADP*_—σ_*inh*_ plane, having ~10 times lower ranges in both directions.

The linear array, see Figure 3A, has again the widest region of non-uniform stationary states. The largest region of stable non-uniform stationary states is the region 3-2, which contains a subregion 3-0 with no stable stationary state delimited by a closed Hopf bifurcation curve, this is an opposite effect to the secondary stabilization by a Hopf bifurcation curve observed earlier. The region 3-2 is further intersected by new pairs of non-uniform solutions arising via symmetry breaking bifurcation curves to form up to 15 stationary states (region 15-4). The regions with three stable non-uniform stationary states represent patterns, where one of them occurs via a secondary symmetry breaking bifurcation. Since the region 9-4 and the region 15-4 intersect the region 9-3 and the region 7-3, two of the stable non-uniform patterns arose from a secondary symmetry breaking bifurcation and therefore they do not have a mirror image as in case of the non-uniform patterns emerging via a primary symmetry breaking bifurcation to form the region 3-2. The T-shaped array is shown in Figure 3B. The widest region 5-2 combines the non-uniform stationary states arising from secondary symmetry breaking bifurcation. The Hopf bifurcation curves (full red curves) destabilize stable non-uniform stationary states, creating closed curves of regions 11-2, 13-2, and 7-0, but also, combined with symmetry breaking curves, they give rise to the region 11-5, where there is the highest number of stable non-uniform stationary states. At *k*_*ADP*_ = 0.02 s^−1^, regions 5-2 and 11-4 further intersects with a symmetry breaking curve adding two new unstable non-uniform stationary states creating the regions 13-4 and 7-2, and further below *k*_*ADP*_ = 0.01 s^−1^, even 15 non-uniform stationary states occur. The cyclic array, shown in Figure 3C, has the narrowest region of non-uniform stationary states, but also contains up to 21 non-uniform stationary states. The largest region 9-2 has 9 stationary states, eight of them arose from a secondary symmetry breaking curve. The Hopf bifurcation curve partly delimits the region 9-2 together with the symmetry breaking and limit point bifurcation curves. The regions 15-4, 15-6, 7-4, 9-4, and 9-5 have such mixed boundaries as well. At *k*_*ADP*_ = 0.02 s^−1^ the region 9-2 and smaller regions are intersected with several symmetry breaking bifurcation curves creating three separate regions 21-6 and other regions with 4 stable non-uniform stationary states.

### Coexistence of Discrete Turing Patterns

**Figure 4:**
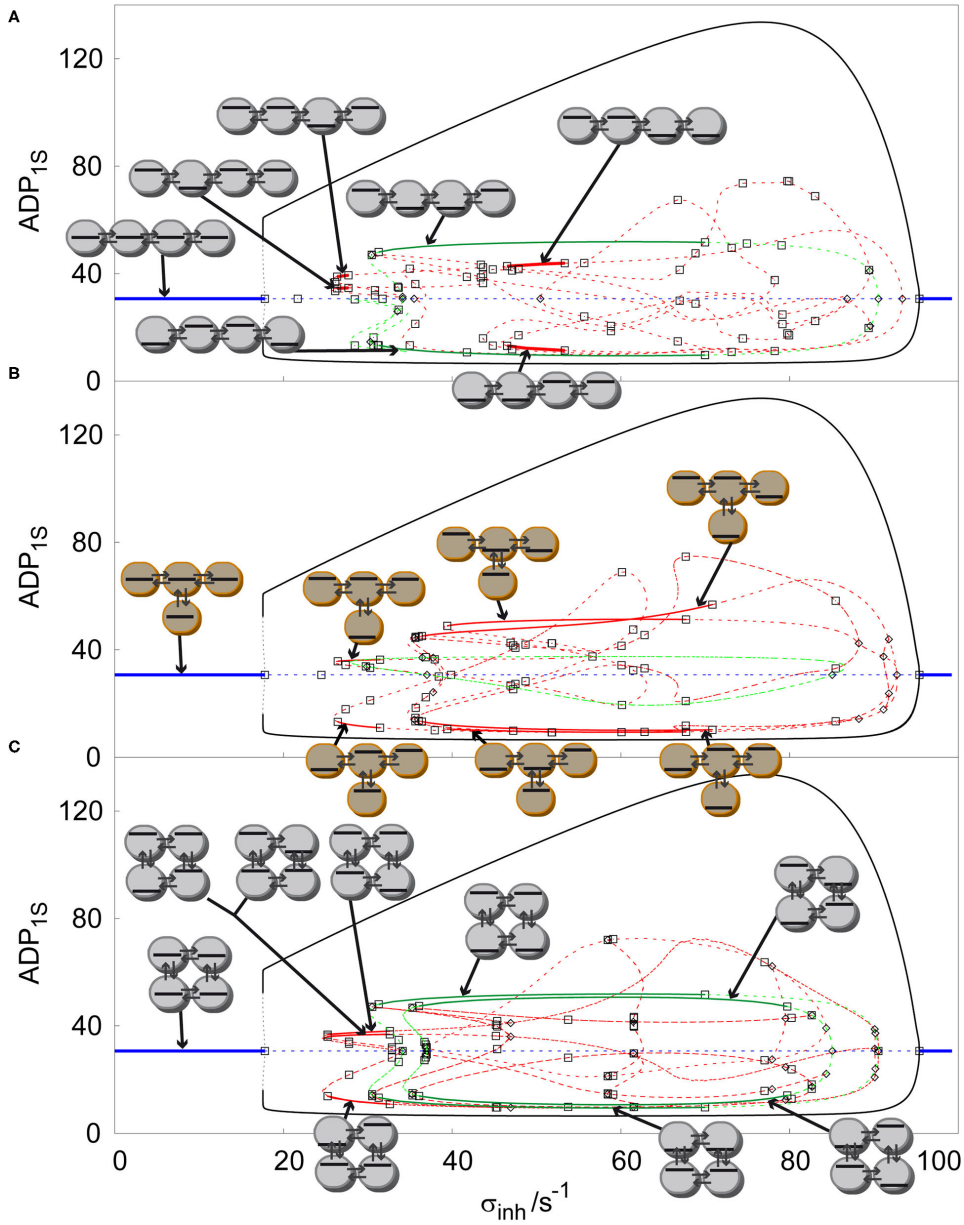
Comparison of solution diagrams of four coupled cells *q* = 100, σ_*M*_ = 100 s^−1^, *k*_*ADP*_= 0.10 s^−1^ in: **(A)** linear array, **(B)** T-shaped array, **(C)** cyclic array. Red curve—asymmetric non-uniform stationary state; blue curve—homogeneous stationary state; green curve—symmetric non-uniform stationary state; full line –stable stationary solution, dashed curve- unstable stationary solution; empty square—Hopf bifurcation point; empty diamond—symmetry breaking bifurcation point. Each stable stationary state has assigned a symbolic representation of its pattern.

After two-parameter analysis of arrays of coupled cells with glycolytic oscillatory reaction, we now focus on one-parameter analysis by creating solution diagrams (also called one-parameter bifurcation diagrams), which are a more suitable tool to distinguish the concentration profiles of all stable non-uniform stationary states (discrete Turing patterns). It is possible to construct any number of solution diagrams associated with the bifurcation diagrams presented in Figures 2, 3 by fixing one of the two parameters in the bifurcation diagram and plotting stationary value of a selected component (ADP in our case) in a selected cell (first cell) against the other parameter. However, we choose to show only representatives for each case by fixing *k*_*ADP*_ at certain value and varying σ_*inh*_ to compare the arrangement of concentration profiles for various arrays. At first, we compare linear, T-shaped and cyclic arrays for *q* = 1, *k*_*ADP*_= 0.1 s^−1^, σ_*M*_ = 100 s^−1^, see Figure 4. As described in section Bifurcation Scenarios SUSS regions are present in every cell in every topology of an array for *q* ≤ 1, which is apparent when comparing all three topologies in Figure 4. The SUSS regions are marked by blue line [the plotted stationary value of ADP is independent of σ_*inh*_ due to specific form of Equation (2)]. The other shared feature are uniform oscillations represented by curves for minima and maxima (black curve) merging at two primary Hopf bifurcation points. The Hopf bifurcation at the right is supercritical, therefore there is a transfer of stability from the SUSS to the SUO. The left Hopf bifurcation point is subcritical and thus the emerging uniform oscillations are unstable (dashed black curve) and only upon a rapid increase of amplitude they become stable via fold bifurcation (full black curve) marking the left boundary of SUO (the corresponding value of σ_*inh*_ is virtually indistinguishable from that corresponding to the Hopf bifurcation).

All stable non-uniform patterns are assigned a symbolic pictographic representation. By increasing σ_*inh*_ in the linear array, Figure 4A, after the Hopf bifurcation point giving rise to uniform oscillations, when following the line of unstable uniform stationary states, there is a primary symmetry breaking bifurcation point, which gives rise to two unstable symmetric non-uniform stationary states (green curve), breaking thus the uniform symmetry. These two unstable symmetric non-uniform stationary states become stable via secondary Hopf bifurcations, which delimit a broad window of two stable Turing patterns (full green lines). When we follow the bifurcated upper unstable symmetric non-uniform branch, a secondary symmetry breaking bifurcation point occurs creating two new non-uniform stationary states, which are asymmetric (red curve), therefore breaking the non-uniform symmetry. When σ_*inh*_ is decreased the unstable asymmetric non-uniform stationary state becomes secondarily stabilized by a Hopf bifurcation point and again destabilized by another Hopf bifurcation point, forming the left narrow window of two stable asymmetric Turing patterns (full red curve). A similar scenario occurs on the right when σ_*inh*_ is decreasing past the upper primary Hopf bifurcation point ultimately forming a second wider window of stable asymmetric Turing patterns overlapping with the window of stable symmetric Turing patterns.

The T shaped array is shown in Figure 4B. The primary symmetry breaking bifurcation on the line of unstable uniform stationary states also creates a symmetric non-uniform stationary state (green curve) which, unlike in the previous case does not become stabilized.On the other hand, it bifurcates further via a secondary symmetry breaking bifurcation to form unstable asymmetric non-uniform stationary states, which are further stabilized to form three separate windows of stable distinct Turing patterns delimited by pairs of secondary Hopf bifurcation points. While the leftmost window is narrow, the two others are broad and overlapping. The cyclic array in Figure 4C displays a symmetry breaking bifurcation from the unstable uniform stationary states from both left and right side of the diagram. Due to the symmetry of the array, this bifurcation gives rise to both symmetric and asymmetric branches simultaneously. The six emerging non-uniform stationary states are unstable but become stabilized by secondary Hopf bifurcations ultimately forming a window of four coexisting stable asymmetric Turing patterns and a much broader window of two coexisting stable symmetric patterns. These windows partly overlap. When further increasing σ_*inh*_ there is another primary symmetry breaking bifurcation point creating a pair of unstable symmetric states with a higher degree of symmetry (see the pictograms), which again become stabilized and form the rightmost broad window delimited by secondary Hopf bifurcation points. The two windows of stable Turing patterns with different symmetry strongly overlap.

**Figure 5:**
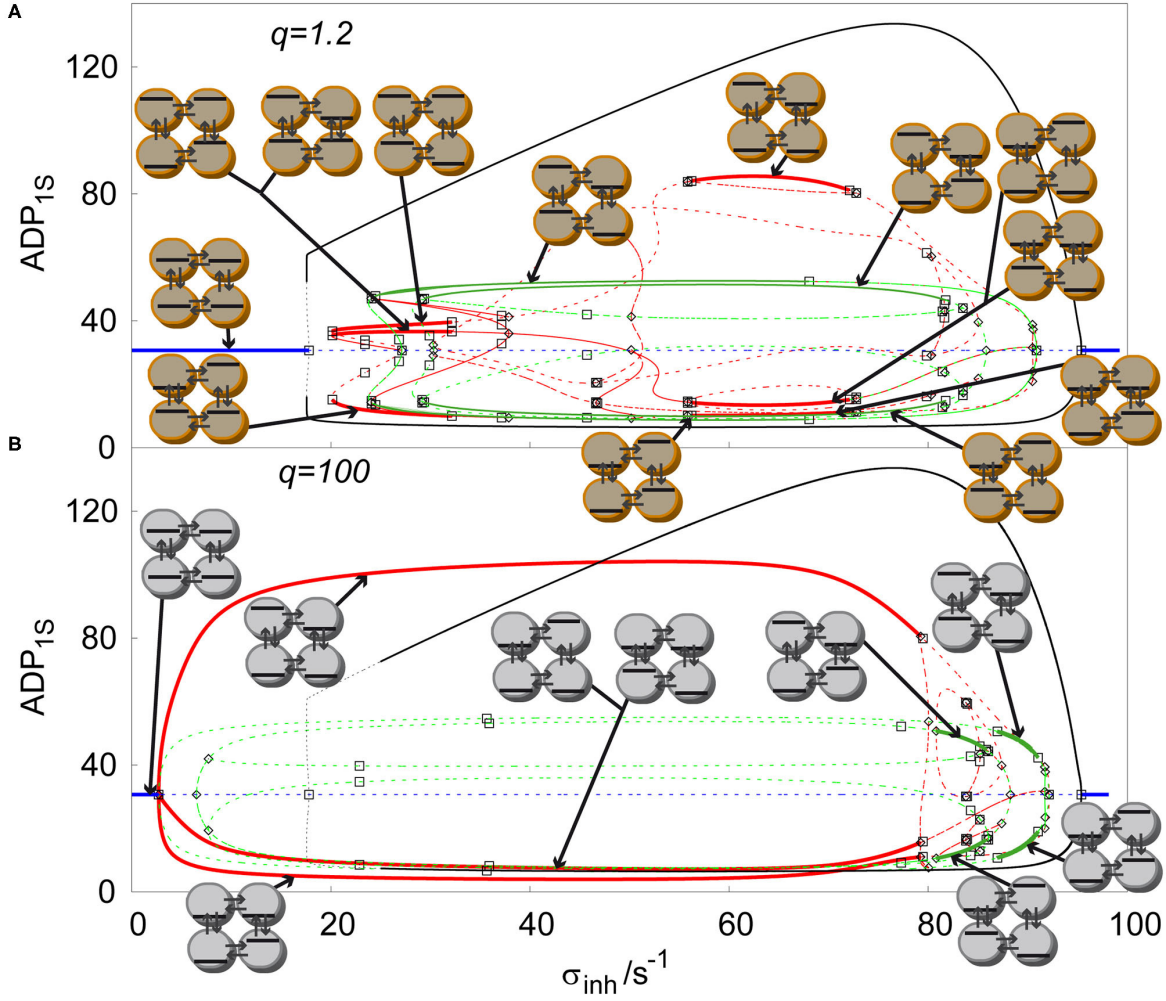
Comparison of solution diagrams of four coupled cells in cyclic array, σ_*M*_ = 100s^−1^, *k*_*ADP*_= 0.10 s^−1^, with varied ratio of *q*: **(A)** *q*=1.2, **(B)** *q* = 100. Red curve—asymmetric non-uniform stationary state; blue curve—homogeneous stationary state; green curve—symmetric non-uniform stationary state; full line—stable stationary solution, dashed curve—unstable stationary solution; empty square—Hopf bifurcation point; empty diamond—symmetry breaking bifurcation point. Each stable stationary state has assigned a symbolic representation of its pattern.

The cyclic array of four coupled cell shows the largest variety of stable non-uniform patterns. It is a promising example of evolution of Turing patterns in a cyclic array system with varying ratio of transport rate coefficients *q*, see Figure 5. The cyclic array with *q* = 1 is shown in Figure 4C. For *q* = 1.2, Figure 5A, the windows of stable symmetric non-uniform stationary states are only slightly altered. When comparing stable asymmetric non-uniform stationary states curve in Figure 5A with Figure 4C, the left window is significantly broader. However, on the right a broad window of stable asymmetric non-uniform stationary states occurs, which is not present for *q* = 1. For *q* = 100, Figure 5B, the curves in the diagram are altered significantly including their stability. The left window of stable asymmetric patterns disappeared entirely and at the same time the other window of stable asymmetric patterns that newly occurred for *q* = 1.2 vastly expanded to the left and covers now almost entire range of non-uniform stationary states. In contrast, the two windows of stable symmetric patterns shifted to the right, became much narrower and do not overlap, neither mutually, not with the window of stable asymmetric patterns. Thus, the effect of varying *q* is profound as already indicated in Figure 3.

## Chemical Computing Devices

The bifurcation analysis in Sections Bifurcation Scenarios and Coexistence of Discrete Turing Patterns shows regions of stable Turing patterns in each system of arrays of cells. To perform chemical computing tasks, the system needs to be parametrically set to the specific regions of coexistence of multiple Turing patterns. To model dynamics of switching between Turing patterns, we incorporated perturbation elements in the Equations (1, 2), specifically:

(3) $$p_{i}\left(t\right)=\{\begin{array}{cc} A_{i} & t_{k}\leq t<t_{k}+\Delta T\;\\ 0 & t_{k}+\;\Delta T\leq t<t_{k+1} \end{array}\;k=0,1,2,\ldots ,m;\;i=1,\ldots ,N.$$

The function *p*_*i*_*(t)* represents a sequence of perturbations in reactor *i* applied by imposing a constant inflow/outflow rate *A*_*i*_, within a time length Δ*T*. *A*_*i*_ represents the amplitude (positive or negative) of perturbation by the species *x* (ATP), applied at times given by *t*_*k*_. A sequence {*t*_*k*_} of *m* perturbations is carefully chosen to avoid effect of previous perturbations to toggle between discrete Turing patterns and oscillations, and also to fit into a clock time given by the *central knockout* system, see section Logic Gates. Specific perturbation amplitudes *A*_*i*_ and time length Δ*T* will be chosen for each type of the chemical computing system. Furthermore, we assume that the level of *y* (ADP) is monitored in each cell to evaluate the state of the system, because stationary state of y does not depend on σ_*inh*_. The concentration *y* of uniform stationary state in one cell is used as a concentration *threshold level* assigning to a non-uniform concentration profile in each cell either logic 1, when the concentration is above threshold level or logic 0, when the concentration is below threshold level.

### Tautology and Contradiction

**Figure 6:**
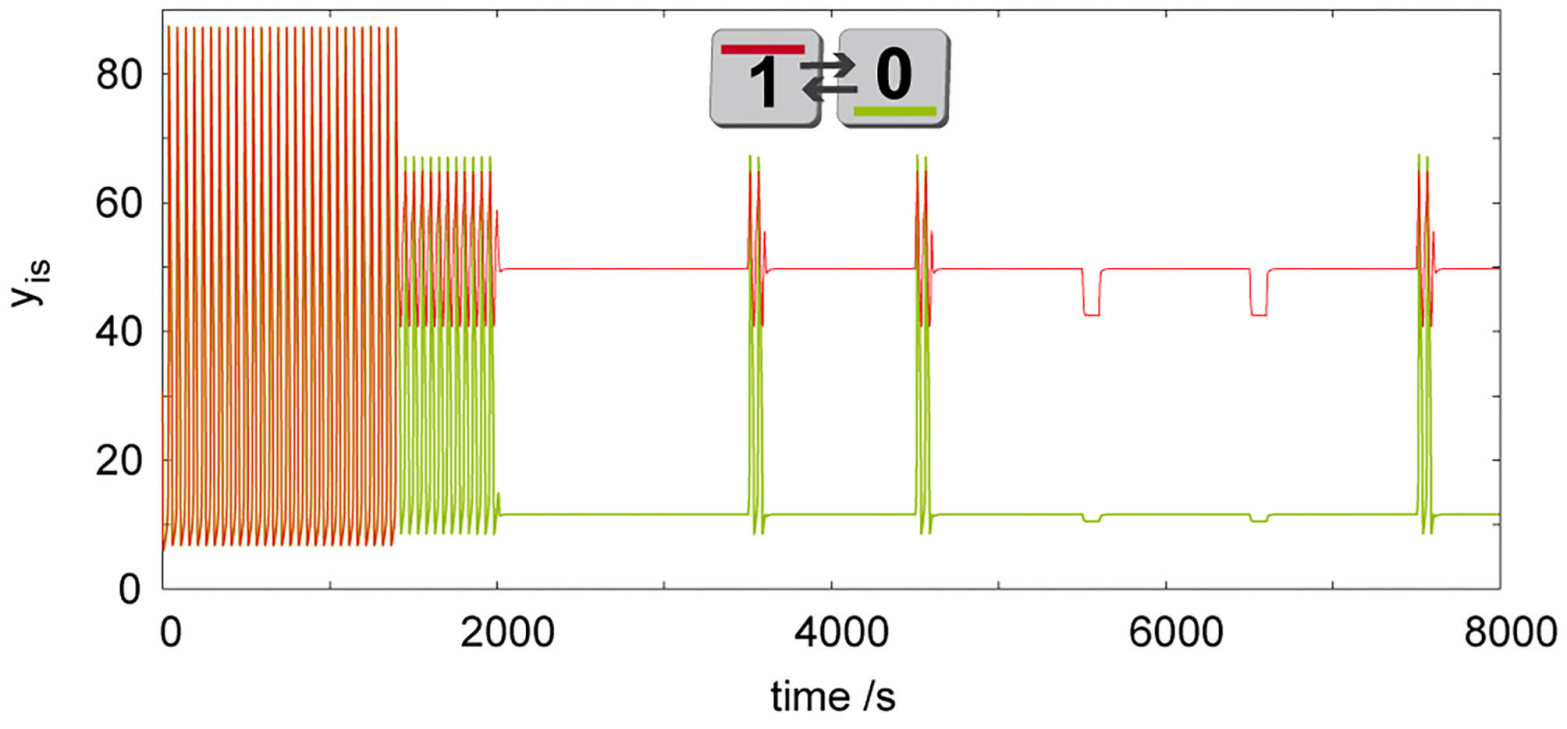
Simulation of dynamic behavior of two cells (*q* = 1, σ_*M*_= 100 s^−1^, σ_*inh*_= 35 s^−1^, *k*_*ADP*_= 0.1 s^−1^) working as a tautology/contradiction gate, where discrete Turing pattern is forced by a long time perturbation (ΔT = 600s, *A*_*i*_ = 1.2) of only a single cell. To show robustness of the discrete Turing pattern, system is further perturbed in the first cell by perturbations *A*_*i*_ = 1.2 at *t*_*k*_= {3,500, 4,500, 7,500} s and by *A*_*i*_ = −0.5 at *t*_*k*_= {5,500, 6,500} s using Δ*T* = 100 s. The only pattern in this figure is shown by its symbolic representation followed by digital 0/1 output assignment.

In our previous chemical computing system (Muzika and Schreiber, 2013; Muzika et al., 2014), simultaneous perturbations with Δ*T* = 100 s are used, which seems to be the minimum perturbation length to achieve transitions under given model parameters (the system shows large amplitude oscillations with the shortest period *T* = 50.29 s). There are also small amplitude oscillations, which are visible through superposition with large amplitude oscillations, having period *T* ≈ 400 s. When the system is carefully perturbed by a positive perturbation for a proper time length Δ*T*, it is possible to induce discrete Turing patterns by perturbation of only one cell in the case of a non-cyclic array. There are numerous possibilities to induce discrete Turing patterns in two coupled cells. An example of such a perturbation using Δ*T* = 600 s applied at time *t*_*k*_ = 1,400 s with *A*_*i*_= 1.2, is shown in Figure 6. We have found that Δ*T* and *t*_*k*_ are mutually dependent when Δ*T* = (2000 – *var*) s, *t*_*k*_ = (100 + *var*) s, for *A*_*i*_ =1.2, where *var* is a time length to be chosen by the user. It is also possible to induce a discrete Turing pattern by certain combination of multiple short perturbations of only one cell, because when only one cell is perturbed, oscillations in all cells start again after the perturbation ends. In this case the system is able to remember previous perturbation for a certain time. Due to this system “memory” discussed in (Goldbeter and Moran, 1984) the induction of discrete Turing patterns is possible using two positive perturbations with the time delay between both perturbations ≤ 500 s. In a linear array of three coupled cells, discrete Turing patterns can be induced by a combination of negative (*A*_*i*_ = −1.0) and positive (*A*_*i*_ = 2.0) perturbations.

Prior to a scheme for advanced cellular assemblages for chemical computing, it is necessary to have a device always defining truth and false statements. In Boolean terminology, we are talking about tautology, which gives always the output true/1 and contradiction, which gives always the output false/0. We propose this device using two coupled cells, where we induce discrete Turing pattern by a perturbation of one cell as shown in Figure 6. The induced Turing pattern is resistant to positive perturbation *A*_*i*_ ≤ 1.2 and negative perturbation *A*_*i*_ ≥ −0.5 therefore such a device can be used as a tautology function in the first cell and a contradiction function in the second cell. In a system composed of advanced cellular assemblages, it can be used as the basic true/false device necessary for knockout perturbations (Muzika and Schreiber, 2013; Muzika et al., 2014), see sections Logic Gates, Advanced Cellular Assemblages Design.

### Logic Gates

Arrays of coupled cells with glycolytic oscillatory reaction show coexistence of non-uniform stationary states and oscillations, see Sections Bifurcation Scenarios, Coexistence of Discrete Turing Patterns. When parameters are set to the region of coexistence of multiple discrete Turing patterns, an array of coupled cells operating under such conditions can be used for chemical computing, provided it is combined with a microfluidic system with carefully targeted perturbations.

Our chemical computing technique, employing digital ones and zeros, is based on transitions among discrete Turing patterns, therefore we need to avoid oscillatory behavior, which does not correspond to digital 1 or digital 0 in this technique. In previous work we proposed a *local knockout* perturbation system (Muzika and Schreiber, 2013; Muzika et al., 2014) to induce a transition from stable uniform oscillations to a user predefined discrete Turing pattern. The local knockout perturbation in those systems was applied 200 s after indication of oscillations using Δ*T* = 100 s.

As a technique more suitable to control larger number of cells in the arrays, we propose a modification of the local knockout perturbation system to a system called the central knockout perturbation system. The difference is that the central knockout perturbation system is designed to control only timings of the knockout perturbation and is triggered at exact times in a row *t*_*k*_= {1,000 *k* + 600} s, *k* = 0, 1, 2, …, *max*, where *max* represents the finite number of perturbation sequences. For three coupled cells using all variations of input perturbations *max* = 8, for four coupled cells using all variations of input perturbations max = 16. Therefore, the central knockout perturbations define a clock rate for chemical computing cells, the current setup has one calculation per one cell per 1000 s.

**Figure 7:**
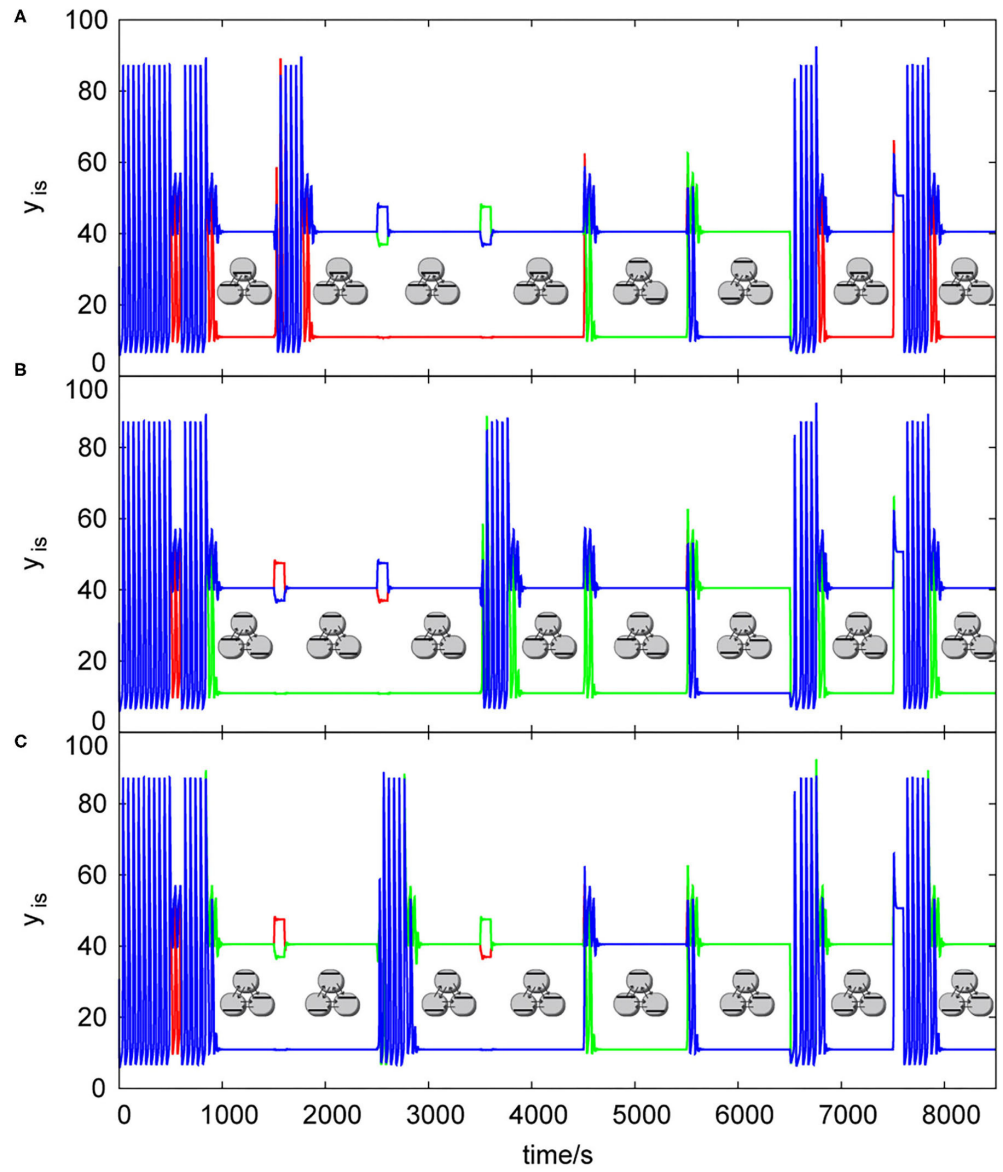
List of dynamic simulations of three coupled cells in cyclic array using central knockout perturbations system, *k*_*ADP*_= 0.1 s^−1^, σ_*inh*_= 35 s^−1^, *q* = 1: **(A)** knockout perturbation {0 1 1}—resulting truth table Table 2B, **(B)** knockout perturbation {1 0 1}– resulting truth table Table 3A, **(C)** knockout perturbation {1 1 0}– resulting truth table Table 3B. Each pattern in this figure is shown by its symbolic representation.

When the central knockout system is activated it sends a signal to the array of cells, see Figure 7, to apply user defined knockout perturbation. The exact knockout perturbation in each array of cells is unique for the array type with specific values of the perturbation amplitudes *A*_*i*_, the perturbation length Δ*T* and also the time delay. The central knockout perturbation system allows each array to calculate a logical function by its own internal parameters, it opens valves for input signals in a form of 1/0 and output valves in form of 1/0 using time delays, *t*_*input*−*delay*_= −100 s and *t*_*output*−*delay*_ = +900 s. Each cell has a receptor similar to the one in local knockout perturbation system. It responds when y_is_ >80 by giving a signal to the knockout valve. When the signal from the receptor and the signal from the central knockout system occur simultaneously, channels are opened according to user settings and the knockout perturbation is applied. Therefore, each knockout valve can be considered as a simple internal AND gate working on a different principle than our knockout logic gate technique.

The knockout perturbation sequence can be set at any time, each array of cells have its unique sequences, which are able to induce a discrete Turing pattern. The user can choose the sequence by creating temporary barriers in the excitable channels (using any excitable channel technique e.g., that used by Górecka and Górecki, 2006) blocking 0 or 1 signal to each cell from tautology/contradiction array, which are not desired for knockout, therefore only proper knockout sequence will be sent into the array. The influence of knockout perturbations on the behavior of the patterns in a cyclic array of three coupled cells is shown in Figure 7. Knockout sequences for three coupled cells in cyclic array are {0 1 1}, {1 0 1}, and {1 1 0}. Each sequence directly influence the type of function calculated in each cell, because it sets the 0 or 1 as the output value instead of oscillations. In Figure 7A, cells are oscillating from time = 0 s until the input perturbation |0 1 1|, at time = 500 s with the time length Δ*T* = 100 s. Then the transition to a discrete Turing pattern occurs until it is perturbed at time = 1,500 s by the input perturbation |1 0 0|, which leads to uniform oscillations. Oscillations were detected and knockout perturbation {0 1 1} was applied approximately at time 1,720 s with the time length Δ*T* = 100 s leading to a discrete Turing pattern at time ~1,840 s. The same sequence of tasks happened with input perturbations |0 0 1| and |0 1 0| until the time 4,500 s, where the input perturbation |1 0 1| with the time length Δ*T* = 100 s leads to a second discrete Turing pattern. At the time 5,500 s, the input perturbation |1 1 0| is applied with the time length Δ*T* = 100 s, which leads to a third discrete Turing pattern. At the time 6,500 s, the input perturbation |0 0 0| is applied which leads to uniform oscillations. They are again knocked out to the user predefined discrete Turing pattern using the knockout sequence {0 1 1}. The same knockout sequence is repeated with the last input perturbation |1 1 1|. Figure 7B shows the same process using input perturbation sequences in Table 2A, but for the knockout sequence {1 0 1}. Figure 7C shows again the same process as in Figure 7A using input perturbation sequences in Table 2A, with the knockout sequence {1 1 0}.

Output functions for each cell in the array assigned to a certain knockout perturbation sequence are described below.

### Truth Tables

To determine the output dynamics to specific input perturbation sequences we added the knockout subroutine to the dynamic simulation program (Kubíček and Marek, 1983; Kohout et al., 2002) and thus we were able to determine response dynamics of arrays of cells and based on these results we were able to determine the output function of each cell and summarize the results in the form of truth tables. To properly describe functions in our truth tables, we express logical functions through their Boolean expressions (Boole, 1854) shown in Table 1. The output functions differ when using the local and central knockout perturbation system with the same kinetic parameter settings. Here we show only the results for the central knockout perturbation system, because it allows us to propose larger computing constructs.

**Table 1:** Two-input logic gates and one-input inverter NOT.

**Logic gate**	**Algebraic Boolean expression**
AND	*A*_1_∙*A*_2_
OR	*A*_1_+*A*_2_
NOT	$\bar{A_{1}}$
NAND	$\bar{A_{1}∙A_{2}}$
NOR	$\bar{A_{1}+A_{2}}$
XOR	$A_{1}⊕A_{2}=A_{1}∙\bar{A_{2}}+\bar{A_{1}}∙A_{2}$
XNOR	$\bar{A_{1}⊕A_{2}}=\bar{A_{1}}∙\bar{A_{2}}+A_{1}∙A_{2}$

In three coupled cells in a cyclic array, the highest number of coexisting discrete Turing patterns is found in the parameter range σ_*inh*_ ≈ (25;40) s^−1^ and *k*_*ADP*_ ≈(0.04;0.15) s^−1^, therefore specific parameters σ_*inh*_= 35 s^−1^, *k*_*ADP*_ = 0.1 s^−1^ used also in refs. (Muzika and Schreiber, 2013; Muzika et al., 2014) fit into this parameter region. We assume the kinetic parameters to be held constant throughout the assemblage of arrays by maintaining the temperature, concentrations of positive and negative effectors, and the pH level. The table of input perturbation sequences for the array of three coupled cells is shown in Table 2A. Dynamical response to these input perturbation sequences in the cyclic array of three coupled cells when using a given knockout perturbation can be viewed as behavior of logic gates responding with output functions to input perturbation sequences. The truth tables for each cell in the cyclic array of three coupled cells responding to the knockout perturbation sequence {0 1 1} is shown in Table 2B. The truth tables for the same array when using the knockout perturbation sequence {1 0 1} is shown in Table 3A, and tables for the knockout perturbation sequence {1 1 0} is shown in Table 3B. By comparing all three truth tables, we can see that the functions ${\bar{A_{1}∙A}}_{2}+A_{3}$ and $A_{1}+\bar{A_{2}∙A_{3}}$ are present simultaneously for two different knockout perturbation sequences.

**Table 2:** **(A)** Table of input signals valid for three coupled cells in cyclic array. **(B)** Truth table for the logic gate with knockout perturbation {0 1 1} (three coupled cells in cyclic array) σ_*inh*_ =35 s^−1^, *k*_*ADP*_ = 0.1 s^−1^.

**(A)**			**(B)**		
**Input signals**			**Output signals**		
**1st cell** **(A_1_)**	**2nd cell** ** (A_2_)**	**3rd cell** ** (A_3_)**	**1st cell** ** (y_1_)**	**2nd cell** ** (y_2_)**	**3rd cell** ** (y_3_)**
0	1	1	0	1	1
1	0	0	0	1	1
0	0	1	0	1	1
0	1	0	0	1	1
1	0	1	1	0	1
1	1	0	1	1	0
0	0	0	0	1	1
1	1	1	0	1	1
			*A*_1_∙(*A*_2_⊕*A*_3_)	$\bar{A_{1}∙A_{3}}+A_{2}$	${\bar{A_{1}∙A}}_{2}+A_{3}$

**Table 3:** **(A)** Truth table for the logic gate with knockout perturbation {1 0 1} (three coupled cells in cyclic array) σ_*inh*_ = 35 s^−1^, *k*_*ADP*_= 0.1 s^−1^. **(B)** Truth table for the logic gate with knockout perturbation {1 1 0} (three coupled cells in cyclic array) σ_*inh*_=35 s^−1^, *k*_*ADP*_=0.1 s^−1^.

**(A)**			**(B)**		
**Output signals**			**Output signals**		
**1st cell** **(y_1_)**	**2nd cell** **(y_2_)**	**3rd cell (y_3_)**	**1st cell** **(y_1_)**	**2nd cell ** **(y_2_)**	**3rd cell ** **(y_3_)**
0	1	1	0	1	1
1	0	1	1	1	0
1	0	1	1	1	0
1	0	1	1	1	0
1	0	1	1	0	1
1	1	0	1	1	0
1	0	1	1	1	0
1	0	1	1	1	0
$A_{1}+\bar{A_{2}∙A_{3}}$	(*A*_1_⊕*A*_3_)*A*_2_	$\bar{A_{1}∙A_{2}}+A_{3}$	$A_{1}+\bar{A_{2}∙A_{3}}$	$\bar{A_{1}∙A_{3}}+A_{2}$	(*A*_1_⊕*A*_2_)∙*A*_3_

The truth tables for four coupled cells with different topology of arrays, different *q* and σ_*inh*_, and for different knockout perturbations are shown in Table 4. As the functions in four coupled cells in the cyclic array repeat, for a given preset knockout perturbation, the resulting functions are shown for each cell based on the value of knockout perturbation applied to each cell. For example, when the knockout perturbation is set to {1 1 0 0} for *q* = 1 and σ_*inh*_= 50 s^−1^, then the resulting logic gate yields $\bar{\left[\bar{\left(A_{1}⊕A_{3}\right)}⊕A_{4}\right]}+A_{1}+\bar{A_{3}+A_{4}}$ in the first cell, $\bar{\left[\bar{\left(A_{1}⊕A_{3}\right)}⊕A_{4}\right]}+A_{2}+\bar{A_{3}+A_{4}}$ in the second cell, $\left(A_{1}⊕A_{2}⊕A_{4}\right)∙A_{3}∙\bar{A_{1}∙A_{2}}$ in the third cell and $\left(A_{1}⊕A_{2}⊕A_{4}\right)∙A_{4}∙\bar{A_{1}∙A_{2}}$ in the fourth cell.

**Table 4:** Table of output functions in each cell.

	**Output signals**			
**Array type**	**1st cell** ** (y_1_)**	**2nd cell** ** (y_2_)**	**3rd cell** ** (y_3_)**	**4th cell** ** (y_4_)**
**T-shaped**, *q* = 1 {0 0 1 0}, *σ_*inh*_* = 35 s^−1^	$A_{1}∙\bar{A_{2}}∙\bar{A_{3}+A_{4}}$	Tautology	$A_{2}+A_{3}+\bar{A_{1}⊕A_{4}}$	$\bar{A_{1}}∙A_{4}∙\bar{A_{2}+A_{3}}$
T-shaped, *q* = 1 {1 0 0 0}*, σ_*inh*_* = 35 s^−1^	$A_{1}+A_{4}+\bar{A_{2}⊕A_{3}}$	Tautology	$\bar{A_{1}+A_{4}}∙\bar{A_{2}}∙A_{3}$	$\bar{A_{1}}∙A_{2}∙\bar{A_{3}+A_{4}}$
**T-shaped**, *q* =1 {0 0 0 1}, *σ_*inh*_*=35 s^−1^	$A_{1}∙\bar{A_{2}}∙\bar{A_{3}+A_{4}}$	Tautology	$\bar{A_{1}+A_{4}}∙\bar{A_{2}}∙A_{3}$	$A_{2}+A_{4}+\bar{A_{1}⊕A_{3}}$
**Linear**, *q* = 1 {0 1 1 0}, *σ_*inh*_* = 35 s^−1^	$A_{1}∙A_{3}∙\bar{A_{2}+A_{4}}$	${\bar{A_{1}∙A}}_{3}+A_{2}+A_{4}$	The same as 2nd cell	The same as 1st cell
**Linear**, *q* = 1 {1 0 0 1}, *σ_*inh*_* = 35 s^−1^	$A_{1}+A_{3}+\bar{A_{2}∙A_{4}}$	$\bar{A_{1}+A_{3}}∙A_{2}∙A_{4}$	The same as 2nd cell	The same as 1st cell
**Cyclic**, *q* = 1, *q* = 1.2 {0 0 1 1} {0 1 1 0} {1 1 0 0} {1 0 0 1}, *σ_*inh*_* = 35 s^−1^	**0** ~$A_{1}∙\bar{A_{3}}∙\left(A_{2}⊕A_{4}\right)$	**0** ~$\left(A_{1}⊕A_{3}\right)∙A_{2}∙\bar{A_{4}}$	**0** ~$\bar{A_{1}}∙A_{3}∙\left(A_{2}⊕A_{4}\right)$	**0** ~$\left(A_{1}⊕A_{3}\right)∙\bar{A_{2}}∙A_{4}$
	**1** ~$\bar{A_{1}}+A_{3}+\bar{A_{2}⊕A_{4}}$	**1** ~$\bar{A_{1}⊕A_{3}}+\bar{A_{2}}∙A_{4}$	**1** ~$\bar{A_{1}}+A_{3}+\bar{A_{2}⊕A_{4}}$	**1** ~$\bar{A_{1}⊕A_{3}}+\bar{A_{2}}∙A_{4}$
**Cyclic**, *q* = 1 {0 0 1 1} {0 1 1 0} {1 1 0 0} {1 0 0 1}, *σ_*inh*_* = 50 s^−1^	**0** ~$\left(A_{2}⊕A_{3}⊕A_{4}\right)∙A_{1}∙\bar{A_{2}∙A_{3}}$	**0** ~$\left(A_{1}⊕A_{3}⊕A_{4}\right)∙A_{2}∙\bar{A_{1}∙A_{3}}$	**0** ~$\left(A_{1}⊕A_{2}⊕A_{4}\right)∙A_{3}∙\bar{A_{1}∙A_{2}}$	**0** ~$\left(A_{1}⊕A_{2}⊕A_{4}\right)∙A_{4|}∙\bar{A_{1}∙A_{2}}$
	**1** ~$\bar{\left[\bar{\left(A_{2}⊕A_{3}\right)}⊕A_{4}\right]}+A_{1}+\bar{A_{2}+A_{3}}$	**1** ~$\bar{\left[\bar{\left(A_{1}⊕A_{3}\right)}⊕A_{4}\right]}+A_{2}+\bar{A_{3}+A_{4}}$	**1** ~$\bar{\left[\bar{\left(A_{1}⊕A_{2}\right)}⊕A_{4}\right]}+A_{3}+\bar{A_{1}+A_{2}}$	**1** ~$\bar{\left[\bar{\left(A_{1}⊕A_{2}\right)}⊕A_{3}\right]}+A_{4}+\bar{A_{1}+A_{2}}$
**Cyclic**, *q* = 1.2 {1 1 0 0} {1 0 0 1} {0 1 1 0} {0 0 1 1}, *σ_*inh*_* = 50 s^−1^	**0** ~$A_{1}∙\bar{A_{3}}∙\left(A_{2}⊕A_{4}\right)$	**0** $\sim \left(A_{1}⊕A_{3}\right)∙\;A_{2}∙\bar{A_{4}}$	**0** ~$\left(A_{2}⊕A_{4}\right)∙\bar{A_{1}}∙A_{3}$	**0** ~$\left(A_{1}⊕A_{3}\right)∙\bar{A_{2}}∙A_{4}$
	**1** ~$\bar{A_{1}}+A_{3}+\bar{A_{2}⊕A_{4}}$	**1** ~$\bar{A_{1}⊕A_{3}}+\bar{A_{2}}∙A_{4}$	**1** ~$\bar{A_{1}}+A_{3}+\bar{A_{2}⊕A_{4}}$	**1** ~$\bar{A_{1}⊕A_{3}}+\bar{A_{2}}∙A_{4}$
**Cyclic**, *q* = 1.2 {1 1 1 0} {1 1 0 1} {1 0 1 1} {0 1 1 1} {1 1 0 0} {1 0 0 1} {0 1 1 0} {0 0 1 1}, *σ_*inh*_* = 30 s^−1^	**0** ~$\bar{A_{2}∙A_{3}∙A_{4}}∙\left(A_{2}+A_{3}+A_{4}\right)∙A_{1}$	**0** ~$\bar{A_{1}∙A_{3}∙A_{4}}∙\left(A_{1}+A_{3}+A_{4}\right)∙A_{2}$	**0** ~$\bar{A_{1}∙A_{2}∙A_{4}}∙\left(A_{1}+A_{2}+A_{4}\right)∙A_{3}$	**0** ~$\bar{A_{1}∙A_{2}∙A_{3}}∙\left(A_{1}+A_{2}+A_{3}\right)∙A_{4}$
	**1** ~$\bar{A_{2}∙A_{3}∙A_{4}}∙\;\left(A_{2}⊕A_{3}⊕A_{4}\right)+\bar{A_{2}+A_{3}+A_{4}}+A_{1}$	**1** ~$\bar{A_{1}∙A_{3}∙A_{4}}∙\;\left(A_{1}⊕A_{3}⊕A_{4}\right)+\bar{A_{1}+A_{3}+A_{4}}+A_{2}$	**1** $\sim \bar{A_{1}∙A_{2}∙A_{4}}∙\;\left(A_{1}⊕A_{2}⊕A_{4}\right)+\bar{A_{1}+A_{2}+A_{4}}+A_{3}$	**1** ~$\bar{A_{1}∙A_{2}∙A_{3}}∙\left(A_{1}⊕A_{2}⊕A_{3}\right)\;+\bar{A_{1}+A_{2}+A_{3}}+A_{4}$

*0~–knockout perturbation has value 0 in the respective column resulting to following function*.

*1~–knockout perturbation has value 1 in the respective column resulting to following function*.

*In order to calculate each function properly, negative operators (NOT, NAND, NOR, NXOR) need to be processed first, otherwise commutative, associative and parenthesis rules apply*.

### Advanced Cellular Assemblages Design

Simple arrays of cells can be used to calculate basic logic functions or to perform regulatory and analysis tasks. A group of arrays of coupled cells can be arranged to form a cellular assemblage capable of advanced chemical computing using its parallel thread potential.

**Figure 8:**
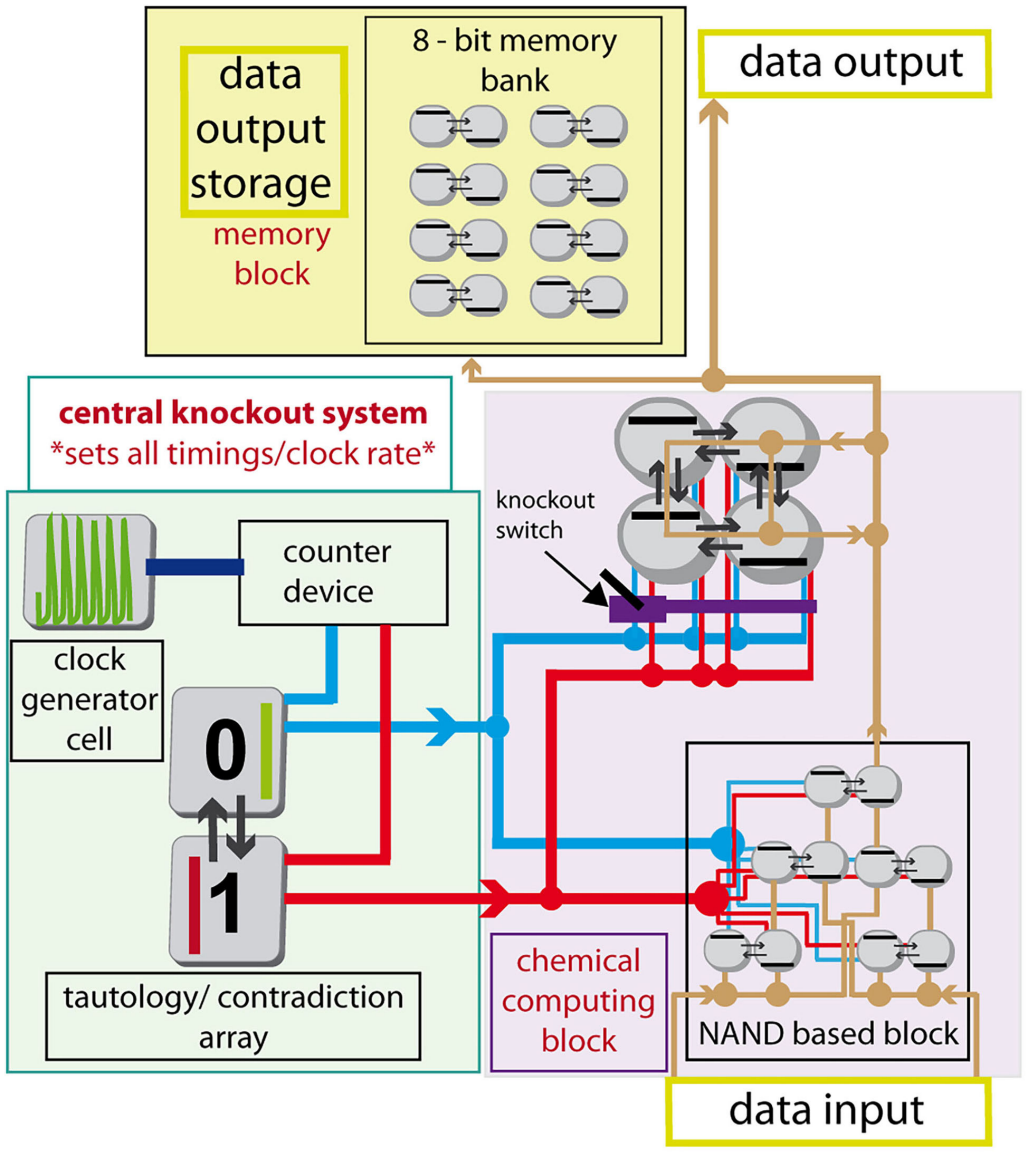
Schematic diagram of an advanced cellular assemblage with chemical computing block, memory block, and central knockout system. Red line—knockout channel with signal 1, blue line—knockout channel with signal 0, brown line—data channel conducting signals 1/0, violet structure -knockout switch, counter device –counts 20 times the period 50.3 s, then sends the pulse into tautology/contradiction device. Full circle on data or knockout channels represents a splitting node.

By assembling several arrays of cells to form an advanced design, connecting outputs of each cell to an adjacent cell of another array as an input perturbation via excitable channel technique and connecting each array to the central knockout perturbation system using again excitable channel technique, we would like to describe, how such an assemblage would work using the tautology/contradiction device, see Figure 8. The excitable channels system can be formed using gel media with immobilized enzymes or immobilized cells performing glycolysis, set to a parameter region of excitation or hard excitation (Goldbeter and Moran, 1984; Bagyan et al., 2005; Bolyó et al., 2010) in a similar way proposed by Górecka and Górecki (2006). The assemblage is divided into three parts: (1) the central knockout system; (2) chemical computing block; (3) memory block. The central knockout system is formed using one cell oscillating naturally with T ≈ 50.3 s; the counter device (similar to Górecki et al., 2003), which stacks up to 20 counts, and then sends a pulse to the tautology/contradiction device while resets itself; and lastly the tautology/contradiction array, see section Tautology and Contradiction, which sends 0 and 1 pulses as a response to the counter device pulses. The whole central knockout perturbation system sends both 1 and 0 signals every ~1,000 s into the system through red and blue channels. The signals carried through channels split at nodes marked by full circles (other crossings of red or blue lines do not correspond to splitting or other interaction). The chemical computing block is proposed as an example and can be formed from completely different types and sizes of arrays of coupled cells. In Figure 8, it is formed using NAND based block (Muzika et al., 2014) without switchable knockout perturbation sequences and block of arrays of four coupled cells in a cyclic array with switchable (user defined) knockout perturbation sequences. The perturbation can be set using a knockout valve. In place of the cyclic 4-array there can be any other array of cells depending on the desired data output. The layout of the NAND based block resembles a subgroup of the layout of CMOS NAND in the 8-bit ALU processor unit. This specific choice does not account for the fact that the 0 signal (blue channel) to every left cell in the mass-coupled pairs () needs to be inverted, neither does it optimize the layout or use the parallel computing power. It is a simple example, the optimization of NAND cells layout is not the goal of this work. The third part is a memory block, which can be built using some of our previously proposed techniques. Specifically, we consider a technique, where 1-bit is stored using perturbation of an array of two cells with the perturbation sequence either |1 0| or |0 1|, details are described in our previous work (Muzika and Schreiber, 2013; Muzika et al., 2014). The advanced cellular assemblage system can work continuously by being perturbed with input perturbation sequences of 1 and 0 (“data input”), while it continuously calculates output stream in a form of 1 and 0. Data stream is conducted through brown channels. Output sequences can be stored in the memory block, used further as “data output” or both operations simultaneously The knockout switch blocks the knockout signal so that only a desired central knockout perturbation sequence will reach the array of cells.

## Discussion and Conclusions

We analyzed three coupled cells in a cyclic array and four coupled cells in linear, cyclic, and T-shaped arrays by methods of one-parameter continuation, two-parameter continuation, and dynamic simulation. We constructed and discussed bifurcation diagrams for all the analyzed systems with a focus on stable non-uniform stationary states (discrete Turing patterns). We performed dynamical simulations to determine response to the central knockout perturbation system with the aim of using them for chemical computing tasks.

By comparing three and four coupled cells with equal ratio of transport rate coefficients of both species ATP and ADP for *q* = 1, we found that discrete Turing patterns occur in arrays of four coupled cells in a wider parameter range than in the case of three coupled cells. When comparing different topology of arrays of four coupled cells, the cyclic array has both the widest parameter regions of occurrence of non-uniform stationary states and the largest number of different discrete Turing patterns. Parameter setting for occurrence of discrete Turing patterns for *q* = 1 can only be found under specific conditions, such as increased temperature, increased pH (Deville-Bonne et al., 1991; Tlapak-Simmons and Reinhart, 1998) and the presence of carbonates (Hereng et al., 2014). Under common laboratory conditions (~25°C, pH~5.5), discrete Turing patterns can only occur in specific media or using specific membranes, where q >> 1. The complexity of discrete Turing patterns at *q* = 100 [the transport rate coefficient of the inhibitor (ATP) is 100 times higher than the transport rate coefficient of the activator (ADP)], is qualitatively similar to discrete Turing patterns at *q* = 1, the difference is in spontaneous vs. non-spontaneous occurrence of patterns, when parameters are varied and also in the range of parameters for which the patterns exist. Another observation we made is that cyclic arrays of cells offer richer selection of Turing patterns, which might benefit the morphogenesis (Turing, 1952).

Analysis of solutions diagrams of all studied arrays of coupled cells shows parameter ranges of coexistence of multiple discrete Turing patterns. Careful transitions between discrete Turing patterns and uniform oscillations using precisely targeted perturbations can be used to design chemical computing devices (Muzika and Schreiber, 2013; Muzika et al., 2014). Using ATP as a signaling species might seem unconventional, as there is ATP in most of the living cells and it might cause interference. Nevertheless, some parts of human brain utilize ATP as a neurotransmitter (Verderioa and Matteolia, 2011) and there are channels with selective permeability to ATP (Locovei et al., 2006), therefore it makes the use of ATP plausible. Our previous technique for the knockout perturbation was focused on oscillations occurring in a specific array. In this work, we propose a modified technique, which creates knockout signal and sends it periodically to each array of cells in an advanced cellular assemblage. Also, it is only applied when the array is currently oscillating. Using this type of knockout technique, the user can switch between functions, which each array utilizes for chemical computing even during the process. Since it controls the computing rate of the advanced cellular assemblage, we call it the central knockout perturbation system.

In a number of papers Katz et al. proposed techniques based on working with enzymes in microfluidic cells using ATP and NAD^+^/NADH as input/output signals and measuring their response in cuvettes using a UV-Vis spectrophotometer. The basic reactor technique is the AND gate using ATP (Privman et al., 2013a) followed by a network of AND gates working both with ATP and NADH (Privman et al., 2013b). NADH also allows enzymatic 1-bit memory units (one bit per cell), which can be arranged to groups by 8 to store ASCII table characters (Pita et al., 2009) similarly as shown in our advanced cellular assemblage scheme, where we use two cells to store one bit. Their technique also allows the release of NADH from the enzymatic computing device to trigger DNA computing (Mailloux et al., 2015). More complicated gates [switch gate, Fredkin gate, half bit adder, half bit subtractor (Fratto and Katz, 2016; Fratto et al., 2016)] can be constructed using three or more interconnected microfluidic devices, where the result is read from cuvette as collected solution from the microfluidic devices as a concentration of NADH and ABTS or ferricyanide/ferrocyanide. These techniques are somewhat closer to measuring metabolites, mainly NADH metabolically connected to ATP/ADP through glycolytic reaction chain as in our experimental system (Muzika et al., 2016), where the level of NADH concentrations is not the result of triggering one cascade of enzymes in microfluidic devices over another cascade of enzymes, but it is the result of transitions between non-uniform spatiotemporal patterns caused by synergy of enzymatic reactions and diffusion.

Gorecki et al. proposed a technique based on inorganic excitable channels utilizing various patterns of pathways of excitable channels. They constructed diodes (Gorecka and Gorecki, 2005; Gorecka et al., 2007; Igarashi et al., 2008), memory units (Górecki et al., 2009), clock generators (Gorecka and Gorecki, 2005), distance detectors (Bagyan et al., 2005; Górecki et al., 2009), band filter (Górecka and Górecki, 2003), logic gates (Sielewiesiuk and Górecki, 2001), and neuron-like structures (Górecka and Górecki, 2006). Such techniques have a great potential especially due to their universality. We expect that the excitable channel technique by Gorecki et al. can be combined with our chemical computing technique and some devices supplemented with it, provided that an excitable glycolytic reaction medium is available (Goldbeter and Moran, 1984; Bagyan et al., 2005; Bolyó et al., 2010). The main difference between excitable vs. Turing pattern techniques is that their memory unit requires a spatial disc reactor, where the waves are constantly traveling, while our memory unit holds its pattern as long as the cells are properly fed and temperature and pH conditions are maintained.

Our current logic gate technique can also be compared with techniques by Holley et al. (2011) and Górecki et al. (2014). By operating the BZ reaction in two oscillatory regimes, the signal is transported through connections between adjacent droplets (gel disks) and, based on the signal type in the output gel droplet it is either digital 0 or digital 1. Using this technique, Adamatzky et al. were able to construct a diode, NAND and XOR logic gate and 1-bit adder. Their technique requires a larger number of droplets for a single basic logic function compared to the number of coupled cell used in our parallel thread chemical computing technique. On the other hand, their technique does not require knockout perturbation system.

Our future research will focus on experiments with transitions between oscillations and non-uniform stationary states in two cuvettes coupled by peristaltic reciprocal pumping (Muzika et al., 2016) and on a chemical computing technique that does not require knockout perturbations.

## Data Availability Statement

The raw data supporting the conclusions of this article will be made available by the authors, without undue reservation.

## Author Contributions

FM and IS: conceptualization, formal analysis, software, writing—review, and editing. IS: funding acquisition. FM, IS, and LS: investigation and methodology. FM: visualization and writing—original draft. All authors contributed to the article and approved the submitted version.

## Conflict of Interest

The authors declare that the research was conducted in the absence of any commercial or financial relationships that could be construed as a potential conflict of interest.
